# No association of *TP53* codon 72 and intron 3 16-bp duplication polymorphisms with breast cancer risk in Chinese Han women: new evidence from a population-based case–control investigation

**DOI:** 10.1186/s40001-018-0345-6

**Published:** 2018-10-11

**Authors:** Weiming Hao, Xia Xu, Haifeng Shi, Chiyu Zhang, Xiaoxiang Chen

**Affiliations:** 1 Institute of Life Sciences, Jiangsu University, Zhenjiang, China; 2 Pathogen Diagnostic Center, Institut Pasteur of Shanghai, Chinese Academy of Sciences, Shanghai, China; 3 Department of Chemotherapy, Jiangsu Cancer Hospital, Jiangsu Institute of Cancer Research, Nanjing Medical University Affiliated Cancer Hospital, Nanjing, 210009 Jiangsu People’s Republic of China; 4 Department of Gynecologic Oncology, Jiangsu Cancer Hospital, Jiangsu Institute of Cancer Research, Nanjing Medical University Affiliated Cancer Hospital, 42# Baiziting Street, Nanjing, 210009 Jiangsu People’s Republic of China

**Keywords:** *TP53*, Polymorphisms, Breast cancer, Chinese women, Case–control investigation

## Abstract

**Background:**

Many studies have demonstrated that the genetic variants of tumor suppressor gene *TP53* contribute to the prediction of breast cancer risk. However, most of them focused on Europeans and Americans; the investigations about Asians, especially Chinese women, are scarce. Thus, the aim of this study was to explore the influence of *TP53* codon 72 and intron 3 16-bp duplication polymorphisms on the breast cancer risk in Chinese women, especially those from eastern China.

**Methods:**

Blood samples collected from 254 breast cancer patients and 252 healthy female individuals were investigated. Genotypes of the two polymorphisms were determined by direct sequencing and conventional PCR, respectively.

**Results:**

Heterozygous Arg/Pro and homozygous Del/Del were the most frequent genotypes of the two polymorphisms, respectively. Heterozygous Arg/Pro had a higher prevalence in breast cancer cases (*P*_adj_ = 0.10; OR_adj_ = 1.43, 95% CI 0.93–2.18), and no homozygous 16-bp duplication (Ins/Ins) genotype was found in the whole 506 clinical samples. For the distributions of allele and haplotype frequencies, no statistically significant difference was observed between the two groups when multiple (additive, dominant and recessive) genetic models were utilized in the analysis (*P*_adj_ > 0.05).

**Conclusion:**

The results suggested that the two *TP53* polymorphisms did not affect breast cancer risk in Chinese Han women, but the heterozygous Arg/Pro may exist as the possible risk genotype of the codon 72 polymorphism in contrast to the homozygous Arg/Arg and Pro/Pro.

**Electronic supplementary material:**

The online version of this article (10.1186/s40001-018-0345-6) contains supplementary material, which is available to authorized users.

## Background

Breast cancer is the world’s most common and deadly cancer in women. It has been demonstrated that the occurrence of this global disease is associated with not only environmental conditions but also genetic susceptibility [1, 2]. The tumor suppressor gene *TP53*, which is located on 17p13.1 and encodes *p53*, is one of the most significant breast cancer susceptibility genes, as the *p53* protein plays an important role in responding to DNA damage, metabolic stress and oncogene activation [3, 4]. However, many genetic variants, especially single nucleotide polymorphisms (SNPs) in the *TP53* gene, have been identified and reported to be cancer associated, since they can result in the changes of amino acids in the DNA-binding domain, and consequently impact the normal functions of *p53* protein [5]. Of them, the most extensively studied was codon 72 polymorphism (Arg72Pro, rs1042522), which is caused by the substitution of arginine (Arg) to praline (Pro). A lot of studies supported that Arg72Pro had correlation with risk of different kinds of carcinoma, especially breast cancer [6–15], whereas other studies did not support it [16–23]. Another well-studied intronic polymorphism is the 16-bp duplication (rs17878362) in intron 3. It was reported that the 16-bp duplication could lead to lower level of *p53* transcript, which may provide a possible molecular basis for the association with high risk of cancer [24]. Although many studies have explored its effect on susceptibility of cancers and found some positive results [8, 11, 20, 22–26], the conclusion is conflicting as well. There are also some new advances concerning genetic factors including breast cancer risk in Chinese [27–33] or Asian [34–37] population. More importantly, most of the previous studies on this issue focused on Caucasians living in Europe and America; the statistics about Asians, especially Chinese population, are relatively scarce. Therefore, we carried out a population-based case–control study to investigate the possible association between the two *TP53* polymorphisms and breast cancer risk in Chinese Han women.

## Methods

### Study population

A total of 506 unrelated clinical peripheral blood samples including 254 women patients with breast cancer from Jiangsu Cancer Hospital and the other 252 healthy female individuals recruited by Zhenjiang Center for Disease Prevention and Control were collected and detected. All the studied breast cancer cases are sporadic cases, as those belonging to familial cases were excluded based on the Breast Cancer Linkage Consortium criteria (Stratton 1997). The clinicopathological information of the patients and healthy controls is shown in Table 1. This study was approved by the Ethics Committee of Jiangsu Cancer Hospital. Informed consent was obtained from all involved participants and publication.

**Table 1:** Clinicopathological information of breast cancer cases and controls

Group	Clinical data	Number (%)
Controls (*N* = 252, mean age = 41.2 ± 10.9)		
Age (years)	< 50	205 (81.3)
	50–70	45 (17.9)
	> 70	2 (0.8)
Breast cancer cases (*N* = 254, mean age = 49.5 ± 9.9)		
Age (years)	< 50	125 (49.2)
	50–70	125 (49.2)
	> 70	4 (1.6)
Menopausal status	Premenopausal	127 (50.0)
	Postmenopausal	114 (44.9)
	NA	13 (5.1)
Tumor type	*IDC*	221 (87.0)
	*ILC*	9 (3.5)
	Others	12 (4.7)
	NA	12 (4.7)
Tumor stage^a^	I	7 (2.9)
	II	164 (67.8)
	III	40 (16.5)
	IV	3 (1.2)
	NA	28 (11.6)

*IDC* invasive ductal carcinoma, *ILC* invasive lobular carcinoma, *NA* not available

^a^According to the AJCC Cancer Staging Manual (7th Edition)

### Genomic DNA extraction and TP53 polymorphisms detection

Genomic DNA (gDNA) was extracted from the blood samples using QIAamp DNA Blood Mini Kit (QIAGEN, Hilden, Germany). The codon 72 polymorphism was determined through direct sequencing. Genomic DNA of each sample was amplified using the forward primer: 5′-GACCTGGTCCTCTGACTGCTCT-3′ and reverse primer: 5′-TGACAGGAAGCCAAAGGGTGAAGAG-3′. The PCR thermal cycling conditions were pre-denaturation at 94 °C for 2 min; 30 cycles of denaturation at 98 °C for 10 s, annealing at 59 °C for 30 s and extension at 68 °C for 25 s. The 430 bp PCR products were directly sequenced using the ABI 3730*xl* DNA Analyzer instrument (Applied Biosystems) after purification from 1.5% agarose gel. The 16-bp duplication polymorphism was determined by PCR using the forward primer: 5′-CGTTCTGGTAAGGACAAGGGTTGG-3′ and reverse primer: 5′-AAAGAGCAGTCAGAGGACCAGGTC-3′. The reaction conditions were pre-denaturation at 94 °C for 2 min; 30 cycles of denaturation at 98 °C for 10 s, annealing at 59 °C for 30 s and extension at 68 °C for 6 s. Then, PCR products were separated by 4% agarose gel and visualized by Gelred staining. Homozygous wild-type alleles (no duplication, designated Del allele) resulted in a 102 bp fragment, while homozygous variant alleles (16 bp duplication, designated Ins allele) resulted in a 118 bp fragment. Both two fragments (102 bp + 118 bp) were obtained when the genotype was heterozygous Del/Ins. All reactions were performed in a total volume of 25 μL mixture containing 20 ng of gDNA and 0.3 µM of each forward and reverse primer.

### Statistical analysis

Statistical analysis was performed using SPSS version 19.0 (SPSS Inc., Chicago, USA) and the online Hardy–Weinberg equilibrium (HWE) calculator (http://ihg.gsf.de/cgi-bin/hw/hwa1.pl). Chi-square (*χ*^2^) test was used to compare the categorical variables. Statistical significance level was set at 0.05. The odds ratio (OR) with its 95% confidence interval (CI) was calculated through logistic regression analysis (adjusted for age) to measure the association between genotypes of the two polymorphisms and breast cancer risk. Additionally, Armitage’s trend test was also performed to improve the statistical power of this study.

## Results

In this study, the distributions of the genotype frequencies of both codon 72 and intron 3 16-bp duplication polymorphisms among control group (*p* = 0.942 and *p* = 0.407) were within Hardy–Weinberg equilibrium. For the codon 72 polymorphism, the heterozygous Arg/Pro was the most frequent genotype in both groups, and had a higher prevalence in breast cancer cases (*P*_adj_= 0.10; OR_adj_ = 1.43, 95% CI 0.93–2.18). The distributions of Arg and Pro allele frequencies were almost equal in both groups (*P*_adj_= 0.52; OR_adj_ = 1.09, 95% CI 0.84–1.42). Regarding the 16-bp duplication polymorphism, the homozygous Del/Del was the most frequent genotype in both groups and its distribution frequency was significantly higher (approximately eightfold) than that of the heterozygous Del/Ins; no homozygous 16-bp duplication (Ins/Ins) carrier was found in the whole 506 samples. Briefly, no statistically significant difference was obtained when analyzing using multiple genetic models (additive, dominant and recessive models) (*P*_adj_ > 0.05) and Armitage’s trend test (*P*_trend_ = 0.58 and 0.86) (Table 2). In addition, concerning the distributions of the genotype frequencies of the two polymorphisms in breast cancer patients with different clinicopathological features, no statistically significant difference was found as well (*p *> 0.05) (Additional file 1: Table S1).

**Table 2:** Distribution of genotype and allele frequencies of *TP53* codon 72 and 16-bp duplication polymorphisms in breast cancer cases and controls

Polymorphism	Genotype	BC cases	Controls	*P*_adj_ value^a^	OR_adj_^a^ (95% CI)	Armitage’s trend test
Codon 72	Additive model					
	Arg/Arg	66 (26.0%)	82 (32.5%)		Reference	*P*_trend_ = 0.58 OR _trend _= 1.05
	Arg/Pro	149 (58.7%)	123 (48.8%)	0.10	1.43 (0.93–2.18)	
	Pro/Pro	39 (15.3%)	47 (18.7%)	0.72	1.11 (0.63–1.95)	
	Dominant model					
	Arg/Arg	66 (26.0%)	82 (32.5%)		Reference	
	Arg/Pro + Pro/Pro	188 (74.0%)	170 (67.5%)	0.16	1.34 (0.89–2.02)	
	Recessive model					
	Arg/Arg + Arg/Pro	215 (84.7%)	205 (81.3%)		Reference	
	Pro/Pro	39 (15.3%)	47 (18.7%)	0.62	0.88 (0.54–1.44)	
Allele	Arg	281 (55.3%)	287 (56.9%)		Reference	
	Pro	227 (44.7%)	217 (43.1%)	0.52	1.09 (0.84–1.42)	
16-bp duplication	Additive model					*P*_trend_ = 0.86 OR _trend_ = 0.95
	Del/Del	230 (90.6%)	227 (90.1%)		Reference	
	Del/Ins	24 (9.4%)	25 (9.9%)	0.58	0.84 (0.44–1.58)	
	Ins/Ins	0 (–)	0 (–)	–	–	
	Dominant model					
	Del/Del	230 (90.6%)	227 (90.1%)		Reference	
	Del/Ins + Ins/Ins	24 (9.4%)	25 (9.9%)	0.58	0.84 (0.44–1.58)	
	Recessive model					
	Del/Del + Del/Ins	254 (100%)	252 (100%)		Reference	
	Ins/Ins	0 (–)	0 (–)	–	–	
Allele	Del	484 (95.3%)	479 (95.0%)		Reference	
	Ins	24 (4.7%)	25 (5.0%)	0.59	0.84 (0.45–1.57)	

*BC cases* breast cancer cases, *OR* odds ratio, *CI* confidence interval

^a^Adjusted by age

Distributions of haplotypes of the two polymorphisms were further analyzed. For all haplotypes, no matter the most frequent Arg-Del or the rarest Arg-Ins, there was also no statistical significance (*P*_adj_ > 0.05) (Table 3).

**Table 3:** Analysis of haplotype frequencies of *TP53* codon 72 and 16-bp duplication polymorphisms in breast cancer cases and controls

Haplotype	BC cases	Controls	*P*_adj_-value	OR_adj_ (95% CI)
Arg-Del	0.48	0.49		Reference
Pro-Del	0.42	0.41	0.67	1.07 (0.79–1.44)
Arg-Ins	0.05	0.04	0.90	1.05 (0.51–2.17)
Pro-Ins	0.05	0.06	0.65	0.86 (0.45–1.64)

## Discussion

Here, we investigated the relationship between two controversial *TP53* polymorphisms codon 72 and intron 3 16-bp duplication as well as their haplotypes, and breast cancer risk in Chinese Han women. For the codon 72 polymorphism, a lot of studies have reported its contribution to the breast cancer susceptibility in women from different geographic areas and ethnic groups [8, 10, 11, 13]. Particularly, Siddique et al. found a strong correlation between the codon 72 Arg allele and susceptibility to Chinese breast cancer development [13]. In their study, the distribution of genotype frequencies of Arg/Arg, Arg/Pro and Pro/Pro among breast cancer cases was 43.5%, 44.0% and 12.5%, respectively; whereas in control group, this distribution was 35.0%, 45.0% and 20.0%, respectively. In contrast to the high prevalence of homozygous Arg/Arg in the breast cancer cases and low prevalence of homozygous Pro/Pro in the controls, the frequency distribution of heterozygous Arg/Pro was almost equal between the two groups. On the contrary, many studies did not support this kind of positive correlation [16, 19, 22, 23]. For instance, Lum et al. found that the codon 72 polymorphism did not affect general breast cancer risk in Chinese women, but the Arg/Arg homozygote seemed to decrease the cancer risk in the later onset sporadic cases (OR = 0.27, 95% CI 0.08–0.93; adjusted for age), which was inconsistent with Siddique’s findings [16]. However, in this study, the results were a little different from those reported by both Siddique and Lum. The distribution frequencies of both Arg/Arg and Pro/Pro homozygotes in breast cancer cases were lower than those among healthy controls; by contrast, the Arg/Pro heterozygote accounted for a larger proportion in both groups, and was more frequent in breast cancer cases, which implied that it might exist as the possible risk genotype of the codon 72 polymorphism despite that there was not any statistical significant correlation under the three genetic models (Table 2). The association has been proved to be more evident in Caucasians living in Europe and America than Chinese Han women. The heterozygous RP but not homozygous RR which relates to increased incidence of breast cancer indicates that this variation had an extremely tiny influence on the function of TP53. Furthermore, no effect of any genotype of the codon 72 polymorphism on breast cancer cases with different clinicopathological features could be observed in this study as well, no matter the later-onset cases or early-onset cases (*p *= 0.85) (Additional file 1: Table S1). These contradictory results were probably caused by the bias of sample collection, such as smaller sample size of either breast cancer case group (Siddique’s study, *N* = 94) or control group (Lum’s study, *N* = 80) of their studies.

Regarding the 16-bp duplication polymorphism, most of the previous studies accepted that the Ins allele, especially homozygous Ins/Ins, contributed to the increase of breast cancer risk [8, 11, 24–26]. However, we could not find any Ins/Ins homozygote carrier in the total 506 samples, implying that this risk genotype may not affect breast cancer risk in Chinese women because of its extremely low prevalence. In terms of the distribution of heterozygous Del/Ins as well as Ins allele frequencies, no statistical significant difference was found between the two groups (Table 2). These indicate that the intron 3 16-bp duplication is not likely to correlate with the development of breast cancer.

On the other hand, the haplotypes of the two polymorphisms may be another important factor affecting breast cancer risk, since it was reported that they were in strong linkage disequilibrium [38]. Some studies have also gained positive results to support this hypothesis, such as Costa et al. found that the Arg-Ins had correlation with breast cancer risk [11], whereas Osorio et al. found a converse result that the Pro-Del was associated with an earlier age at the onset of the first primary tumor [23]. However, just as the codon 72 polymorphism, which kind of haplotype is indeed the risk factor of the breast cancer progression remains to be seen. In this study, no significant association was found between the haplotypes and Chinese breast cancer risk, no matter the mentioned Arg-Ins, Pro-Del, or the other two kinds of haplotypes (Table 3).

## Conclusion

In conclusion, through a population-based case–control study, we found that there was no significant association between *TP53* codon 72 as well as intron 3 16-bp duplication polymorphisms and breast cancer risk in Chinese Han women when multiple genetic models along with Armitage’s trend test were utilized in the analysis, but the heterozygous Arg/Pro may exist as the possible risk genotype of the codon 72 polymorphism, which were a little distinguished from the conclusions of previous studies. Still, more relevant studies designed for Chinese population, especially those with larger sample size, need to be done to further validate these findings.

## Additional file

**Additional file 1: Table S1.** Distributions of the genotype frequencies of codon 72 and intron 3 16-bp duplication polymorphisms in breast cancer patients with different clinicopathological features.

## Abbreviations

SNPs: single nucleotide polymorphisms
OR: odds ratio
CI: confidence interval
